# Classification of Strawberry Maturity Stages and Varieties Using Neural Networks Based on Volatile Organic Compounds

**DOI:** 10.3390/foods14020169

**Published:** 2025-01-08

**Authors:** Jing Huang, Xuenan Zhang, Hang Yang, Zhenbiao Li, Zhengfang Xue, Qingqing Wang, Xinyuan Zhang, Shenghua Ding, Zisheng Luo, Yanqun Xu

**Affiliations:** 1 College of Biosystems Engineering and Food Science, Key Laboratory of Agro-Products Postharvest Handling of Ministry of Agriculture and Rural Affairs, Zhejiang University, Hangzhou 310058, China; 12113065@zju.edu.cn (J.H.); 22213032@zju.edu.cn (X.Z.); lizhenbiao@zju.edu.cn (Z.L.); 22313083@zju.edu.cn (Z.X.); wqq1998@zju.edu.cn (Q.W.); luozisheng@zju.edu.cn (Z.L.); 2 Innovation Center for Postharvest Agro-Products Technology, Zhejiang University, Hangzhou 310058, China; 3 School of Intelligent Systems Science and Engineering, Jinan University, Zhuhai 519070, China; yanghang@stu.jnu.edu.cn (H.Y.); zhangxy@jnu.edu.cn (X.Z.); 4 Hunan Agricultural Product Processing Institute, Hunan Academy of Agricultural Sciences, Changsha 410125, China; shhding@hotmail.com

**Keywords:** strawberry, maturity, variety, volatile organic compounds, machine learning, neural networks

## Abstract

Volatile organic compounds (VOCs) are closely associated with the maturity and variety of strawberries. However, the complexity of VOCs hinders their potential application in strawberry classification. This study developed a novel classification workflow using strawberry VOC profiles and machine learning (ML) models for precise fruit classification. A comprehensive VOC dataset was rapidly collected using gas chromatography-ion mobility spectrometry (GC-IMS) from five strawberry varieties at four maturity stages (n = 300) and visualized through principal component analysis (PCA). Five ML models were developed, including partial least squares discriminant analysis (PLS-DA), decision trees, support vector machines (SVM), Xgboost and neural networks (NN). The accuracy of all models ranged from 90.00% to 98.33%, with the NN model demonstrating the best performance. Specifically, it achieved 96.67% accuracy for single-maturity classification, 98.33% for single-variety classification, and 96.67% for dual maturity and variety classification, along with 98.09% precision, 97.92% recall, and 97.91% F1 score. Feature importance analysis indicated that the NN model exhibited the most balanced reliance on various VOCs, contributing to its optimal performance with the broad-spectrum VOC detection method, GC-IMS. Overall, these findings underscore the potential of NN modeling for accurate and efficient fruit classification based on integrated VOC profiles.

## 1. Introduction

Strawberry (*Fragaria × ananassa*) is one of the most favored berry fruits worldwide, with an annual production exceeding 10 million tons [1]. However, their economic significance is also closely tied to their perishability and short shelf life, which averages only 3–4 days [2]. This makes accurate maturity and variety determination critical for optimizing harvest timing, postharvest handling, and reducing supply chain losses. Current methods of assessing strawberry maturity primarily rely on visual inspection, such as color changes, which are limited in their accuracy. This often leads to suboptimal harvest timing and high postharvest losses, which can reach up to 50% of the total supply [3]. Similarly, variety identification is typically based on physical traits, which are subjective and prone to error. Given these challenges, a more precise method of maturity and variety classification is needed to improve decision-making in strawberry production and supply chain management.

Aroma plays a key role in strawberry quality, as the fruit’s distinctive scent is a primary factor in consumer preference. The genus Fragaria derives its name from the Latin word “*fragrans*”, meaning sweet-scented, emphasizing the importance of volatile organic compounds (VOCs) in fruit quality [4]. Over 50 VOCs have been identified in strawberries, with key contributors such as hexenals, furanones, and terpenes defining their aroma profile [5]. The composition of VOCs undergoes significant changes during strawberry ripeness stages and different varieties. For example, most esters increase as the fruit matures, while specific compounds such as trans-2-hexanal are predominantly present during the early stages [6]. VOC profiles also vary among different strawberry cultivars, providing a unique chemical fingerprint that reflects the genetic characteristics of the fruit [7]. These distinctive patterns highlight the potential of VOC profiles as a non-destructive tool for classifying fruit ripeness and variety. However, specific environmental conditions and postharvest handling methods may also impact strawberry VOCs [8]. Lv et al. [9] compared the flavor compounds of ripened strawberry (“Sweet Charlie”) stored at 4, 23, and 37 °C. Their results indicated that higher storage temperatures (23 and 37 °C) induced higher levels of esters but lower levels of aldehydes. Yang et al. [10] found that treatments with preservatives such as 1-MCP and ClO_2_ could enhance the content of esters, trans-2-hexenal and volatile ketone 4-hydroxy-2,5-dimethylfuran-3-one (DMHF), contributing to the maintenance of strawberry flavor during storage. Consequently, overcoming the complexity of VOCs in strawberries has emerged as a new challenge for practical application.

In recent years, machine learning has emerged as a powerful tool for food quality prediction due to its ability to process large, multi-dimensional datasets. Models like support vector machines (SVM), neural networks (NN), and decision trees have been successfully applied to various agricultural products, yielding promising results in tasks such as flavor profiling, ripeness classification, and shelf life prediction [11,12,13]. However, for fruits with strong aromas such as strawberries, the strategy of bidirectional integration of machine learning with flavor characteristics for the precise prediction of maturity and variety has not been fully studied. Although an upgraded mathematical model was developed by Matar et al. [14] to predict the shelf life gain of fresh strawberries “*Charlotte cv*” in modified atmosphere packaging, this model can only input limited indicators such as time, temperature and internal gas composition (O2/CO2), but cannot handle a large number of relevant VOCs input elements. Other machine learning algorithms that were compared by Do et al. [15] can be effectively used for linear regression prediction of postharvest strawberry storage period, but they cannot solve complex classification tasks such as strawberry variety identification. The integration of machine learning with VOCs, particularly for agricultural classification tasks, still requires in-depth investigations.

The significance of this study lies in bridging this gap by developing and evaluating a novel VOC-based machine learning workflow specifically for strawberry maturity and variety classification. By comparing the performance of five machine learning models—partial least squares discriminant analysis (PLS-DA), decision trees, support vector machines (SVM), Xgboost and neural networks (NN)—this research aims to identify the most effective model for integrating VOC data into fruit classification systems. This approach aims to enhance the precision of strawberry quality control, thereby minimizing postharvest losses and optimizing supply chain efficiency. Furthermore, the methodology offers valuable insights for the classification and grading of other aromatic fruits, providing a basis for advancing innovative postharvest technologies.

## 2. Materials and Methods

### 2.1. Plant Materials

Five strawberry varieties (*Akihime*, *Benihoppe*, *Ningyu*, *Hongyu*, *Fenyu*) were cultivated in the same greenhouse of Xihu District, Hangzhou City, Zhejiang Province, China. The four different maturity stages were defined as big green, half red, red and overripe according to [16]. Two batches of strawberry samples were collected in this experiment. The first batch was collected in December 2023. Five strawberry fruits were taken as a parallel and 12 parallel groups were picked for each specific variety and maturity, respectively, to conduct subsequent GC-IMS determination and establish the VOC dataset 1(Table S1). This dataset was used for model training and validation with a ratio of 80% to 20% [17]. The second batch, collected in March 2024, consisted of three parallels per specific variety and maturity for subsequent VOC dataset 2 (Table S2) and was used as the final model test dataset. In total, 300 groups of samples were used in this study. All strawberries were harvested from the greenhouse and transported to the laboratory within 2 h. The fruits were then diced into 1 cm × 1 cm cubes and frozen at −80 °C promptly.

### 2.2. GC-IMS Analysis

Analyses of strawberry samples were performed using a FlavourSpec^®^ system (GAS, Dortmund, Germany) equipped with an MXT-5 (15 m × 0.53 mm × 1 μm), an IMS detector, and a headspace autosampler.

Strawberry fruits were ground into fine powder using liquid nitrogen just prior to VOC analysis and then homogenized with a saturated calcium chloride salt solution (1:1, *m*/*v*). Then, 4 mL of the extract was placed in a 20 mL glass headspace sampling vial and its analysis was finished within 2 h to ensure the integrity of VOC composition. The vial was then heated at 40 °C with agitation at 250 rpm for 30 min. A 500 µL volume of resultant headspace was automatically injected into the heated injector (80 °C) with a heated syringe (85 °C). The GC column was maintained at 60 °C (isothermal mode) and the nitrogen carrier gas (≥99.999%) operated at the following programmed flow rate: 2 mL/min for 2 min, then the flow was linearly raised to 10 mL/min within 8 min, then to 100 mL/min within 10 min, and finally the flow reached 150 mL/min within the next 10 min. The sample was then analyzed in positive ion mode using purified nitrogen as the drift gas at 150 mL/min with the drift tube maintained at 45 °C. The retention index (RI) of each compound was calculated using n-ketones C4–C9 (Sinopharm Chemical Reagent Beijing Co., Ltd., Beijing, China) as external references [18] due to its no response between alkanes and IMS. Unknown volatiles were identified by comparing detected RI and the drift time of standards during the GC-IMS library supplied by G.A.S [19].

### 2.3. PCA Analysis

Principal component analysis (PCA) is based on a linear projection of multidimensional data into different coordinates based on maximum variance and minimum correlation [20]. Training patterns from measurements of similar samples will be located close to one another after transformation. Hence, a graphical output can be used to determine the difference between groups and to compare this difference with the pattern distribution within a group [21]. Using PCA, the measurement data were transformed into 3D coordinates through a data reduction, which extracts the most important information. This process was carried out using the web tool MetaboAnalyst (https://www.metaboanalyst.ca/, accessed on 11 October 2024).

### 2.4. Machine Learning Models Construction

#### 2.4.1. Partial Least Squares Discriminant Analysis (PLS-DA)

The train_test_split function was used to split the dataset into a training set and a test set, facilitating both model training and evaluation. PLSRegression was applied to construct two models: the ripeness classification model (pls_ripeness_model) and the variety classification model (pls_variety_model). Feature dimensionality, which refers to the number of features and the complexity of the dataset, here corresponded to 61 components. The number of principal components, which reduces the total number of features through linear combinations of the original features, was ultimately reduced to 10 in this model. Finally, model parameters were optimized through 5-fold cross-validation to ensure optimal classification performance.

#### 2.4.2. Support Vector Machine (SVM)

The SVM model was constructed using Python 3.11 and its sklearn library. To optimize model performance, the regularization parameter C and the kernel type were tuned during the training process. For the kernel function, the radial basis function (rbf) was selected for maturity classification, while the polynomial kernel function (poly) was chosen for variety classification. The regularization parameter was set to 1.0 to balance model complexity and its error penalty. Model parameters were optimized through 5-fold cross-validation to ensure optimal classification performance.

#### 2.4.3. Decision Tree

Python 3.11 and its sklearn library were also used for decision tree construction. The DecisionTreeClassifier, with its default parameters, was directly employed in the model construction process. The parameters included criterion = ‘gini’ for the split criterion, splitter = ‘best’ for the split strategy, max_depth = None (where the maximum depth of the tree is unlimited until all leaves are pure or contain fewer than min_samples_split samples), and min_samples_split = 2 (the minimum number of samples required to split an internal node). Model parameters were optimized through 5-fold cross-validation to ensure optimal classification performance.

#### 2.4.4. Extreme Gradient Boosting (Xgboost)

The Xgboost was implemented using Python 3.11 and its Xgboost library, a tree-based learning algorithm optimized by the gradient boosting framework and widely applied in classification, regression, and other machine learning tasks. The model’s performance was enhanced by optimizing key parameters, resulting in a learning rate of 0.3, maximum tree depth of 6, subsample ratio of 1, and column sampling ratio of 1. In addition, grid search and 5-fold cross-validation methods were employed to ensure optimal generalization capability and predictive accuracy.

#### 2.4.5. Neural Network (NN)

The NN model, based on a multilayer perceptron (MLP) architecture, contains three main components: the input layer, hidden layer and output layer. Among them, the feature number of the input layer changes dynamically according to the feature count of dataset, which is specifically set as 61 in this model, corresponding to the 61 different flavor compounds detected in strawberries. Hidden layers are used to perform nonlinear transformations and feature extraction, capturing complex relationships within the dataset. This model consisted of two hidden layers, the first with 128 neurons and the second with 64 neurons. A ReLU (Rectified Linear Unit) activation function was applied to the hidden layer to introduce nonlinearity. The number of neurons in the output layer was determined by the number of labels in the dataset. In this model, a softmax activation function was used in the output layer to convert the output into a probability distribution, which is beneficial to multi-class classification tasks. Finally, model parameters were optimized through 5-fold cross-validation to ensure optimal classification performance.

### 2.5. Performance Evaluation of the Trained Machine Learning Models

Accuracy, precision, recall and F1 score are four commonly used evaluation indicators for machine learning classification models. To calculate these metrics, it is necessary to construct the confusion matrix which is a table to visualize the algorithm performance [22]. In the context of a multi-classification model, accuracy measures the global sample prediction situation, defined as the ratio of the number of correct predictions to the total number of samples. Precision and recall are calculated separately for each class, and then the weighted-average method (https://scikit-learn.org/, accessed on 15 October 2024) is used to assign different weights to different categories. F1 score is a combination of precision and recall. All these metrics are used to comprehensively evaluate the model performance and need to be calculated by the following formula [13].

$$Accuracy=\frac{TP+TN}{TP+TN+FP+FN}$$

$$Precision=\frac{TP}{TP+FP}$$

$$Recall=\frac{TP}{TP+FN}$$

$$F1\;score=\frac{2\times Precision\times Recall}{Precision+Recall}$$

where:

- TP (True Positive): correctly predicted as positive values because their real value is positive.
- FP (False Positive): incorrectly predicted as positive values because their real value is negative.
- FN (False Negative): incorrectly predicted as negative values because their real value is positive.
- TN (True Negative): correctly predicted as negative values because their real value is negative.

### 2.6. Feature Importance of the Trained Machine Learning Models

Feature importance was calculated using the permutation importance method. Specifically, the values of a specific feature (feature i) in the training dataset were permuted to generate a modified dataset (X_train_permuted), while keeping all other features fixed. The original model was then used to predict with X_train_permuted, and the accuracy score (score_permuted) was calculated. The permutation importance for each feature was the difference in accuracy scores (score_permuted—score).

## 3. Results and Discussion

### 3.1. Preprocessing of the Data

The aroma components of strawberries in different varieties and maturities were detected by GC-IMS, with qualitative analysis based on retention times and drift times. A total of 61 substances were identified from 75 points, mainly including aldehydes, ketones, esters, alcohols, terpenes and furanones (Table S3). These compounds are primarily synthesized through four major VOC biosynthetic pathways: the cytosolic mevalonic acid (MVA) and plastidial methylerythritol phosphate (MEP) pathways which produce terpenoids, the shikimate/phenylalanine pathway which produces phenylpropanoid and benzenoid compounds, and the lipoxygenase (LOX) pathway which generates volatile fatty acid derivatives [23]. Among them, 14 VOCs exhibited both monomer (H) and dimer (D) products, resulting in the same substance being identified at different retention times. These phenomena mostly appeared in relatively high-content components such as (E)-2-hexenal for dimer formation due to their high proton affinity or signals [24].

**Figure 1:**
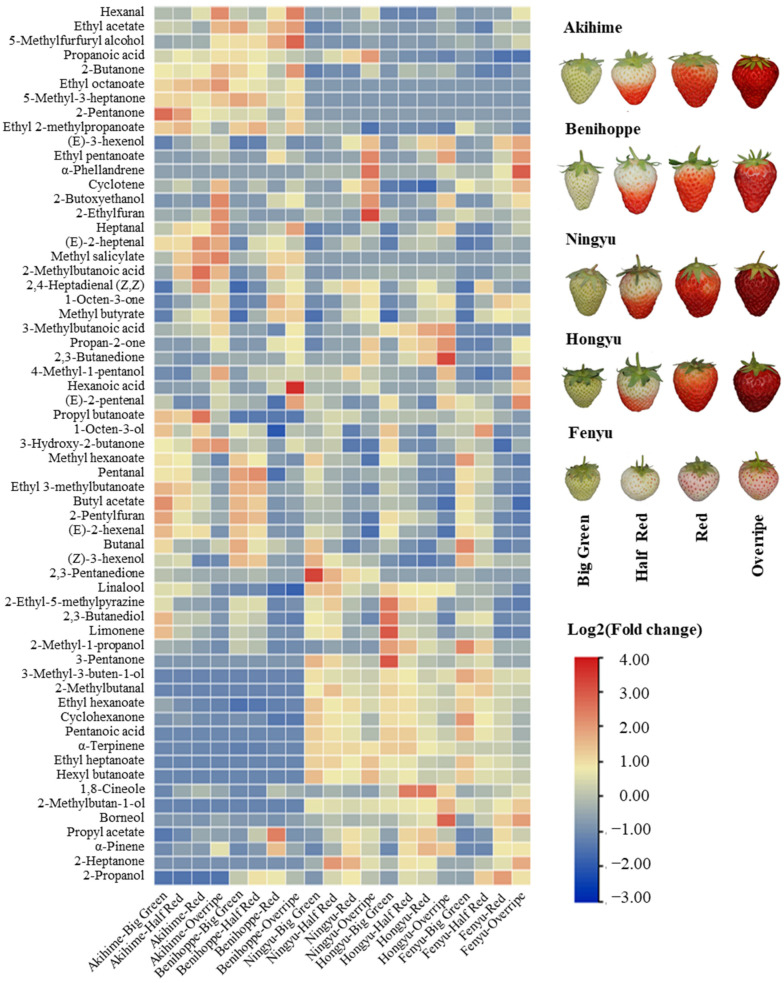
The heatmap of volatiles analysis and fruit phenotype in five strawberry varieties (*Akihime*, *Benihoppe*, *Ningyu*, *Hongyu*, *Fenyu*) at four developmental stages (big green, half red, red, and overripe).

To facilitate comparison, a 2D difference map between different strawberry varieties is shown in Figure S1. The analysis revealed that most compounds had retention times ranging from 200 to 800 s, with relative drift times (DT) from 4 to 8. When the big green plot was selected as the reference, other maturity stages exhibited much more volatiles in almost all strawberry varieties except *Akihime*. Furthermore, as shown in Figure 1, the content of (E)-2-hexenal, butanal and (Z)-3-hexenol showed a downward trend following the strawberry ripening stages, while several esters such as propyl acetate and methyl butyrate, and alcohols such as 2-butoxyethanol that showed an opposite trend. This is consistent with the research results of Padilla-Jiménez et al. [25] that esters are closely related to the aroma of mature strawberries. Several components have also been found with a higher content in specific varieties such as 2,3-pentanedione in *Ningyu* and 1,8-Cineole in *Hongyu* (Figure 1). In addition, 15 characteristic components were identified exclusively in specific strawberry varieties (Table S4). Among them, ethyl octanoate was found only in *Akihime* and *Benihoppe* while 2-methylbutan-1-ol was detected in *Hongyu*, *Ningyu* and *Fenyu*. These differences provide a theoretical basis for distinguishing strawberry varieties and maturities based on VOC profiles.

### 3.2. Visualization of the Data

**Figure 2:**
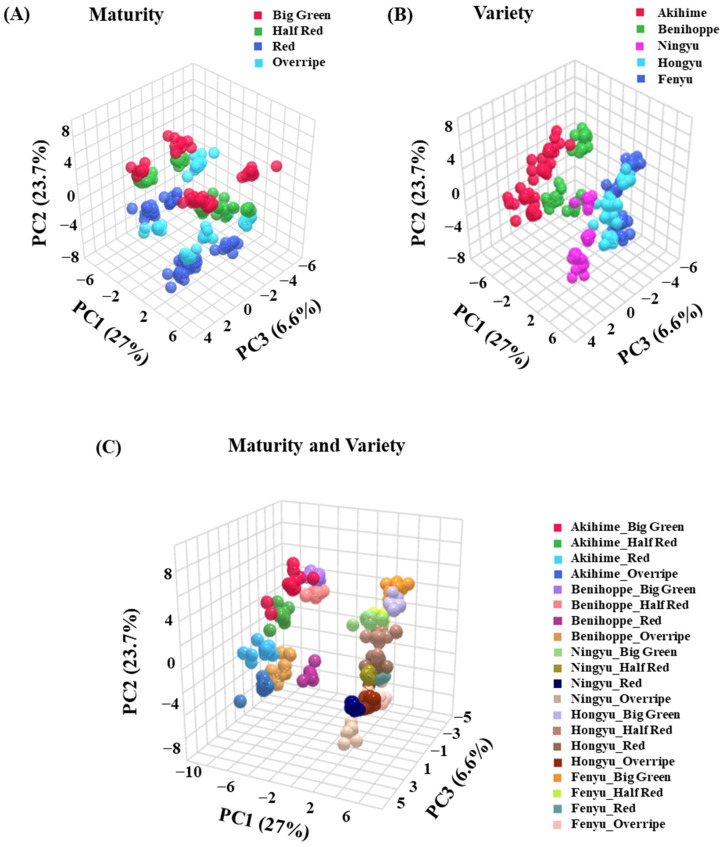
Principal component analysis (PCA) analysis of volatile components in four strawberry maturities (**A**), five strawberry varieties (**B**), strawberry maturities and varieties (**C**).

Understanding data characteristics through visualization is essential before applying suitable analysis techniques. Principal component analysis (PCA) was conducted to reduce dimensionality and identify potential partitions within the dataset [26]. Figure 2 presents the PCA score plots for strawberries at different maturities and varieties based on VOCs. The first three principal components together explained 57.3% of all variance. As for maturity classification, VOCs at different maturity stages can be basically separated though samples within the same group have some differences, resulting in dispersed distributions rather than tight clustering (Figure 2A). However, for variety classification, Figure 2B demonstrated that PCA cannot distinguish the fruits from different cultivars effectively, particularly among the *Hongyu*, *Ningyu* and *Fenyu* groups due to their high similarity. When classified by both variety and maturity, Figure 2C revealed a less distinct separation than a single classification. Groups including the *Fenyu*_Big Green, *Fenyu*_Half Red and *Fenyu*_Red were entirely indistinguishable. Although more principal components (PCs) were analyzed, the first five PCs (PC1-PC5) accounted for only 67.9% of the variance (Figure S2). All these results present that PCA cannot cover the complexity of the dataset well and that it is hard to distribute the dataset. Additionally, PCA is just a dimensionality reduction technology that can convert high-dimensional data into low-dimensional data while retaining some characteristics of the original data [27]. It can be used for result classification and data visualization, but it cannot be directly used for unknown sample prediction [13]. Therefore, to achieve high-quality data classification and prediction of unknown strawberry samples, machine learning techniques were considered to deal with this problem.

### 3.3. Use of Different Machine Learning Models for Classification of Strawberry Samples

**Figure 3:**
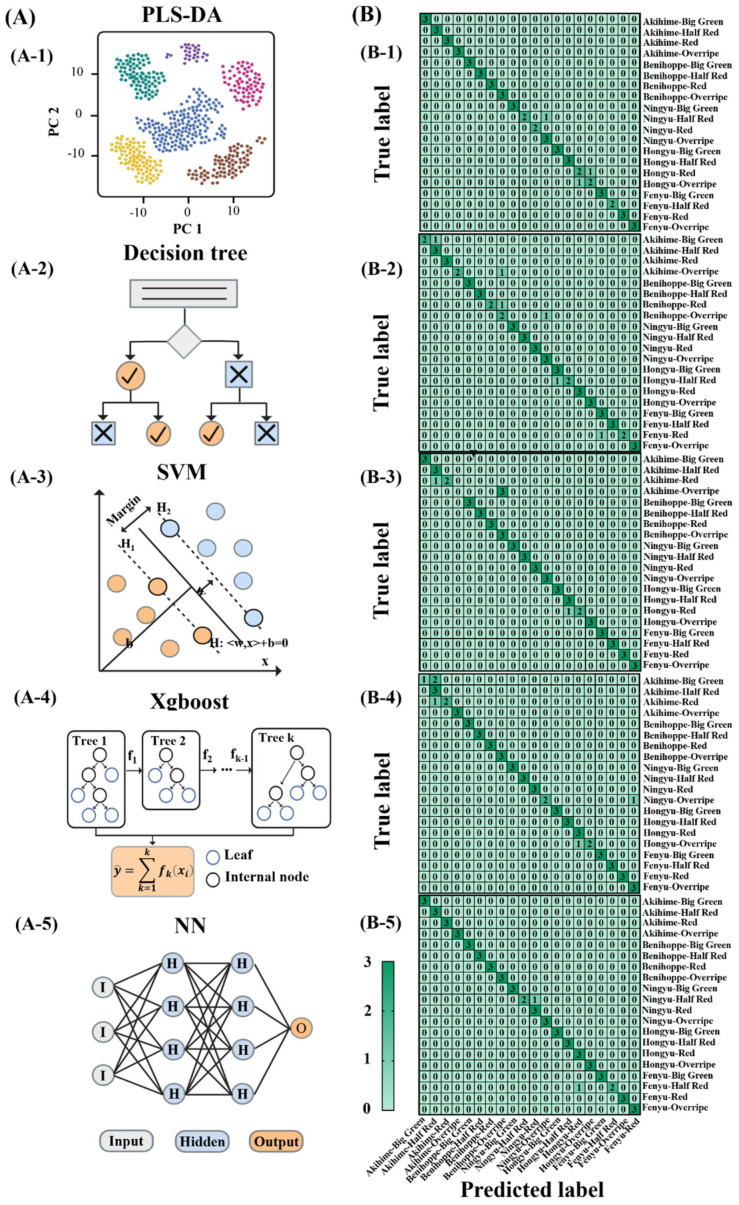
Schematic diagram (**A**) and confusion matrix (**B**) of five machine learning models PLS-DA (**A-1**,**B-1**), SVM (**A-2**,**B-2**), decision tree (**A-3**,**B-3**), Xgboost (**A-4**,**B-4**) and NN (**A-5**,**B-5**) in strawberry maturities and varieties. Four maturities include big green, half red, red, and overripe while five varieties include *Akihime*, *Benihoppe*, *Ningyu*, *Hongyu* and *Fenyu*.

Supervised machine learning techniques have been widely adopted for data classification in various application fields, utilizing input–output datasets from known categories to identify patterns [11]. Each algorithm varies in its approach to model construction, feature learning, and application suitability. PLS-DA could effectively distinguish latent variables across categorical encoding while SVM excels in high-dimensional and nonlinear data analysis by identifying optimal decision boundaries [28,29]. The decision tree offers simplified model interpretation and flexibility through recursive splitting point identification [30]. Xgboost, an ensemble method, achieves superior predictive accuracy by combining decision trees with gradient boosting [31]. Neural network (NN), which is inspired by human brain functionality, excels in complex pattern decoding across large-scale datasets [32]. All these five models were constructed here and applied to the classification of strawberry variety and maturity, aiming to pinpoint the most effective model through comparative performance analysis. The schematic diagrams of those five machine learning models are shown in Figure 3A.

#### 3.3.1. Discrimination of Different Maturities and Varieties

Strawberries emit minimal volatiles when immature but significantly more as they ripen [6]. Zhao et al. [7] also compared the volatile types and contents across different strawberry varieties, confirming that VOCs can serve as indicators for both maturity and variety. Five machine learning models (PLS-DA, SVM, decision tree, Xgboost and NN) were constructed, with their confusion matrices shown in Figure 3. NN was the best model that reached 96.67%, with only individual errors in the predictions of Ningyu_Half Red and Fenyu_Half Red categories. The accuracy of the remaining four models was relatively close (91.67%, 90.00%, 91.67%, 91.67% at PLS-DA, decision tree, SVM and Xgboost), but their error types were different. Among them, false cases predicted by PLS-DA, decision tree and Xgboost were relatively scattered. By contrast, false predictions of SVM were more concentrated, especially for Akihime_Overripe category, which were all misidentified as Benihoppe_Overripe. As shown in Table 1, other evaluation indicators including precision, recall and F1 score were also highest in NN, then followed by Xgboost.

#### 3.3.2. Discrimination of Different Maturities

**Figure 4:**
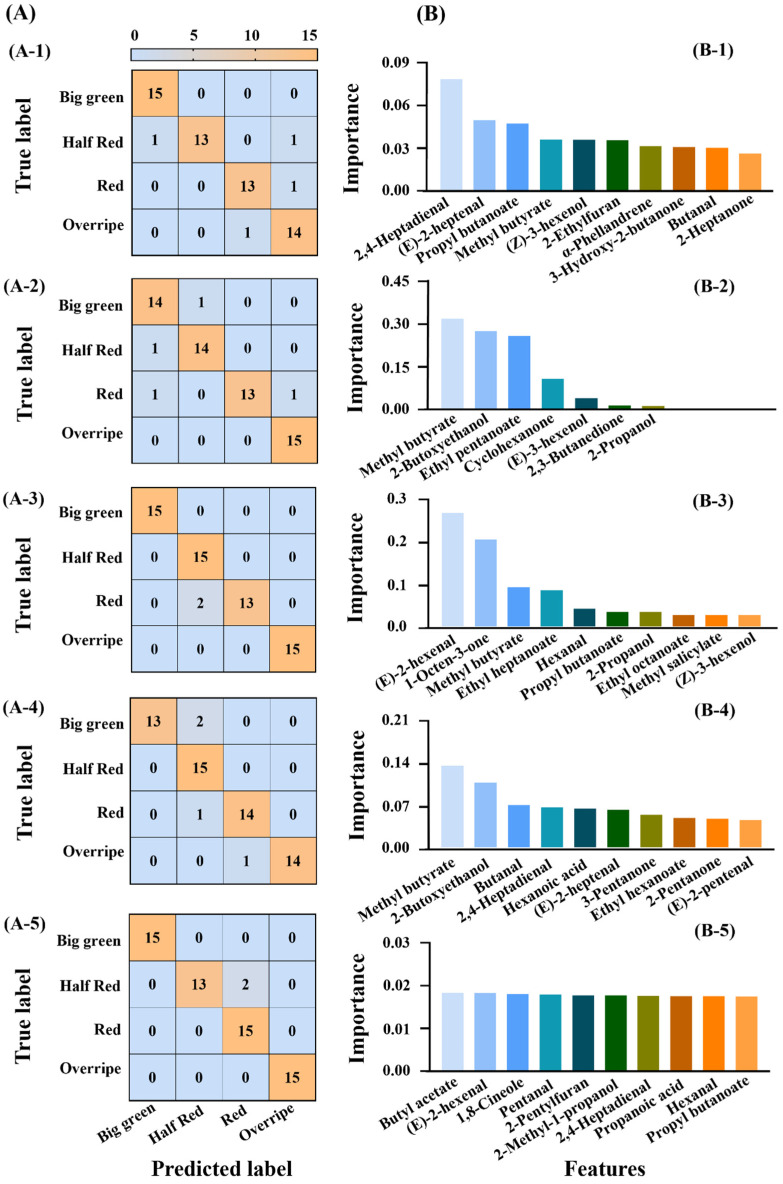
Confusion matrix (**A**), feature importance (**B**) of five machine learning models PLS-DA (**A-1**,**B-1**), SVM (**A-2**,**B-2**), decision tree (**A-3**,**B-3**), Xgboost (**A-4**,**B-4**) and NN (**A-5**,**B-5**) in strawberry maturities (big green, half red, red and overripe). The yellow blocks represent the number of correctly predicted samples while the blue represents the number of incorrectly predicted samples (**A-1–A-5**).

In the above dual classification of strawberry varieties and maturity stages, refining classification categories could reduce the number of samples in each specific group, potentially affecting model performance, which required validation. Therefore, single-direction classification predictions were conducted, focusing separately on strawberry variety and maturity. Confusion matrixes of all models to predict strawberry maturities are shown in Figure 4A. SVM and NN were the best models that reached 96.67% accuracy, with both reporting a few false cases during the red and half-red periods, respectively. Decision tree and Xgboost followed with 93.33%, while PLS-DA was the lowest at 91.67%, exhibiting complete accuracy only in the big green stage but inaccuracies in the other three maturity groups (Table 2). Feature importance was also tested to present the contribution to model explainability of individual features in the training process [33]. The feature importance of each compound for model construction is presented in Table S5, and the top ten VOC features ranked by model importance are presented in Figure 4B, having an important effect on strawberry maturity. The effect of each component was the most balanced in the NN model, while it was relatively concentrated in the decision tree and SVM. Methyl butyrate played an important role in different models, and due to its importance, ranked among the top ten in the other four models except NN.

In addition, other evaluation metrics including precision, which represents the accuracy of relevant instance identification, recall, which represents the model’s ability to capture all relevant instances, and F1 score, which represents the balance between precision and recall [34], were also employed to assess model stability in Table 1. Calculated from the confusion matrices, these evaluation metrics were consistent with the accuracy trends, being the highest in NN and the lowest in PLS-DA, while the prediction scores in SVM, Xgboost and decision tree models were relatively close. Overall, NN presented the best predictive ability for classifying different strawberry maturities, outperforming other models significantly in terms of accuracy, precision, recall, and F1 score.

#### 3.3.3. Discrimination of Different Varieties

**Figure 5:**
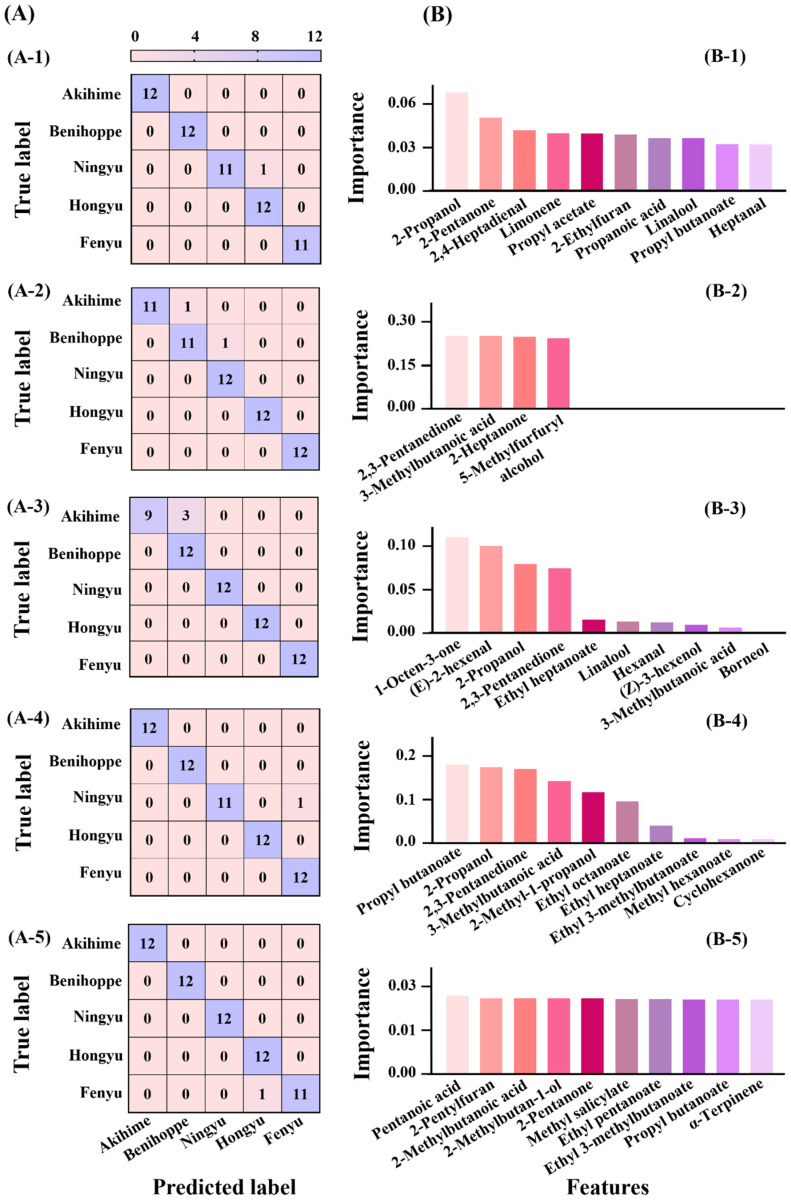
Confusion matrix (**A**), feature importance (**B**) of five machine learning models PLS-DA (**A-1**,**B-1**), SVM (**A-2**,**B-2**), decision tree (**A-3**,**B-3**), Xgboost (**A-4**,**B-4**) and NN (**A-5**,**B-5**) in strawberry varieties (*Akihime*, *Benihoppe*, *Ningyu*, *Hongyu*, *Fenyu*). The purple blocks represent the number of correctly predicted samples while the pink represents the number of incorrectly predicted samples (**A-1**–**A-5**).

For single-direction strawberry variety classification, the confusion matrices are shown in Figure 5A. Xgboost and NN achieved the highest accuracy at 98.33%, followed by PLS-DA and decision tree at 96.67%, and the lowest 95% in SVM (Table 2). Xgboost and NN reported slightly false cases in Ningyu and Fenyu categories but SVM showed more false cases in identifying *Akihime*. PLS-DA reported one false case in *Ningyu* and *Fenyu* while the decision tree misclassified one case in *Akihime* and *Benihoppe*, respectively. These indicated that the feature recognition capability of different machine learning algorithms varies across categories and underscored the necessity to compare models for optimal performance. In feature importance analysis (Figure 5B), NN showed the most balanced contribution of components, similar to the strawberry maturity classification. However, the top ten most important VOC features differed between maturity and variety classifications. For variety classification, 2,3-pentanedione, 3-methylbutanoic acid, 2-propanol and propyl butanoate were important VOCs, as each ranked in the top ten features across three different models. Specifically, 2,3-pentanedione and 3-methylbutanoic acid were present in SVM, Xgboost, and decision tree, 2-propanol in SVM, PLS, and Xgboost, while propyl butanoate in NN, PLS, and Xgboost.

The precision, recall, and F1 scores of the proposed models, calculated from the confusion matrices (Table 1), aligned with accuracy trends, with Xgboost achieving the highest and SVM the lowest values. Notably, although the decision tree exhibited higher accuracy in variety identification (96.67%) than maturity (93.33%), its precision, recall, and F1 scores for variety were much lower than those for maturity identification. This underscored the necessity to combine multiple indicators to evaluate model performances comprehensively [34].

Overall, all those models demonstrated excellent performance in classifying strawberry varieties, with Xgboost and NN being the most effective. The models generally performed better in variety classification than in maturity prediction, which might be attributed to the more pronounced manifestation of VOC feature differences among different varieties. Moreover, although the PLS-DA model was not satisfactory when applied to strawberry maturity classification, it performed excellently in variety prediction, highlighting the importance of selecting the appropriate model for different applications.

During the single classification, the proportion of erroneous predictions in model validation was notably lower than that in the dual classification of strawberry variety and maturity. This can be attributed to the fact that refining categories within a fixed dataset size reduces the sample count per group. The finding aligns with Vabalas et al. [35], who demonstrated that insufficient sample sizes can significantly impair predictive performance. Overall, NN and SVM performed well in predicting strawberry maturities, while NN and Xgboost were better suited for varieties. Considering both variety and maturity differences, NN emerged as the best model for strawberry classification. Compared to other algorithms, the NN model proved to be more computationally intensive, requiring considerably more time for training. While models such as decision tree, SVM, and Xgboost exhibited average execution times ranging from 0.55 to 3.96 s, the NN model took approximately 23.64 s due to its higher complexity. All models were conducted on a computer with an Intel Core i7-12700 CPU and 32 GB of RAM, and the software environment utilized Python 3.11 along with libraries such as pandas, sklearn, and matplotlib. Despite the increased resource requirements, the NN model’s performance benefits outweighed the computational cost for this task. This superiority is likely attributed to several key advantages of the NN model: firstly, its unparalleled ability to capture complex, non-linear relationships within data stems from the layered structure and activation functions; secondly, the NN model excels at feature learning, automatically extracting and generating relevant features from raw data, which allows it to identify subtle patterns that might be missed by other algorithms requiring manual feature engineering; and lastly, the flexibility of NN to be customized and adapted for a wide array of tasks by fine-tuning their architecture and parameters makes it particularly effective for the classification tasks of predicting strawberry varieties and maturities. All these characteristics collectively contribute to the superior predictive performance of the NN model.

Analyzing the feature importance of VOC components across different models revealed two distinct patterns (Figure 4B and Figure 5B, Table S5). In the NN models, the importance values of various components were evenly distributed, indicating a diffuse contribution of each VOC. In contrast, the SVM, Xgboost, and, particularly, the decision tree demonstrated more concentrated importance values. As for the decision tree model, three compounds (methyl butyrate, 2-butoxyethanol, and ethyl pentanoate) were particularly critical for predicting strawberry maturity, with permutation importance values of 0.31, 0.27, and 0.25, respectively. Similarly, four key VOCs (2,3-pentanedione, 3-methylbutanoic acid, 2-heptanone, and 5-methylfurfuryl alcohol) played dominant roles in strawberry variety classification, with values of 0.25, 0.25, 0.25, and 0.24. Commonly, VOC detection instruments can be classified into broad-spectrum and non-broad-spectrum categories. Broad-spectrum devices like GC-MS and GC-IMS can detect a wide range of volatile organic compounds, making them well-suited for complex samples [36]. These instruments could align well with the NN model, where VOC importance is diffusely spread. In contrast, non-broad-spectrum instruments like electronic noses, which rely on specific sensors for detecting particular compounds, exhibit varying sensitivities among their sensors [37]. For instance, metal oxide semiconductor sensors are often more sensitive to alcohols, whereas electrochemical sensors are efficient at detecting aldehydes [38,39]. This specificity makes non-broad-spectrum devices more appropriate for models like decision trees, SVM, and Xgboost, where VOC contributions are concentrated in specific key components. This study proposed a novel machine learning strategy for food VOC detection, which paired the VOC importance patterns in a specific model with its appropriate detection instruments. However, it should be acknowledged that this study primarily investigated the differences in VOC profiles among strawberry varieties and maturity stages. Environmental factors such as storage conditions, temperature and humidity, which are known to influence changes in VOC profiles [8], were not considered in this research. As these factors can introduce variations in VOC profiles, they may consequently affect the accuracy of the machine model’s classification. Therefore, the application of these models requires a comprehensive evaluation of specific conditions and corresponding parameter optimization.

## 4. Conclusions

In this study, five strawberry varieties (*Akihime*, *Benihoppe*, *Ningyu*, *Hongyu*, *Fenyu*) were analyzed at four different stages (big green, half red, red and overripe) by GC-IMS for data collection. A total of 61 VOCs were identified and established the connections with strawberries successfully. These connections encompassed the variations in VOC contents and components, as well as the differences among strawberries of various maturities and varieties. For data visualization, PCA analysis was performed which can basically separate different strawberry maturity classifications but failed in variety classification. Five models, including PLS-DA, decision tree, Xgboost, SVM and NN, were constructed and applied to strawberry classification prediction. Results showed that NN was the best predictive model for the dual classification of strawberry maturity and variety, reaching 96.67% and 98.33% accuracy, respectively. SVM was suitable for single maturity prediction, while Xgboost excelled in variety. According to feature importance analysis, the effects of each VOC component in the NN model were the most balanced, while methyl butyrate and 2,3-pentanedione played critical roles in the other models. The varying VOC importance patterns across different models underscored their distinct adaptability potential for broad-spectrum or non-broad-spectrum VOC detection instruments. Overall, the integration of VOC profiles via GC-IMS detection with machine learning techniques is proven to be an effective strategy for predicting strawberry maturity and variety classifications. Although specific VOC profiles and key markers of other fruits may necessitate parameter refinement to enhance classification accuracy, this study provides a robust foundation for extending VOC-based classification to diverse fruits and agricultural products.

## Figures and Tables

**Table 1:** Performance metrics of strawberry maturity and variety by different machine learning techniques.

		Precision (%)	Recall (%)	F1 Score (%)
PLS-DA	Maturity	89.58	87.50	88.50
	Variety	97.92	95.83	96.83
	Maturity and variety	93.75	91.67	92.66
Decision tree	Maturity	94.78	93.75	93.79
	Variety	92.00	91.67	91.39
	Maturity and variety	93.26	91.67	90.72
SVM	Maturity	94.92	93.75	93.63
	Variety	91.96	89.58	89.60
	Maturity and variety	93.44	91.67	91.61
Xgboost	Maturity	95.45	95.24	95.29
	Variety	99.42	99.40	99.41
	Maturity and variety	97.44	97.32	97.35
NN	Maturity	98.09	97.92	97.91
	Variety	98.09	97.92	97.90
	Maturity and variety	98.09	97.92	97.91

**Table 2:** Accuracy of strawberry maturity and variety by different machine learning techniques.

		Accuracy of Different Models (%)				
		PLS-DA	Decision Tree	SVM	Xgboost	NN
Maturity	Big green	100.00	93.33	100.00	86.67	100.00
	Half red	86.67	93.33	100.00	100.00	86.67
	Red	86.67	86.67	86.67	93.33	100.00
	Overripe	93.33	100.00	100.00	93.33	100.00
Average accuracy of maturity		91.67	93.33	96.67	93.33	96.67
Variety	Akibime	100.00	91.67	75.00	100.00	100.00
	Benihoppe	100.00	91.67	100.00	100.00	100.00
	Ningyu	91.67	100.00	100.00	91.67	100.00
	Hongyu	100.00	100.00	100.00	100.00	100.00
	Fenyu	91.67	100.00	100.00	100.00	91.67
Average accuracy of variety		96.67	96.67	95.00	98.33	98.33

## Data Availability

The original contributions presented in the study are included in the article/Supplementary Materials, further inquiries can be directed to the corresponding author.

## Supplementary Materials

The following supporting information can be downloaded at: https://www.mdpi.com/article/10.3390/foods14020169/s1, Figure S1: Spectrum comparison of the volatile compound profile of five strawberry varieties at four developmental stages. Big green stage was set as the reference plot. The color of the points represents the concentration of the substance (color bar), with white indicating identical concentration, blue indicating low concentration and red indicating high concentration. Figure S2: Principal component analysis (PCA) analysis from PC1 to PC5 of volatile components in four strawberry maturities (**A**) and five strawberry varieties (**B**). Table S1: VOC dataset for machine learning modeling. All values represent the components’ content proportion (%). Table S2: VOC dataset for model testing. All values represent the components’ content proportion (%). Table S3: Qualitative analysis of volatile components in different strawberries. RI: Retention index; RT: Retention time [sec]; DT: Drift time [a.u.]. Table S4: The 15 characteristic components only identified in specific strawberry varieties and their contents proportion (%). Table S5: Feature Importance of five machine models for maturity and variety classification.

## Abbreviations

VOCs: volatile organic compounds; GC-IMS, gas chromatography-ion mobility spectrometry; PCA, principal component analysis; PLS-DA, Partial least squares discriminant analysis; SVM, Support vector machine; Xgboost, Extreme gradient boosting; NN, Neural networks.
