# Supplementary material for: Classification of Strawberry Maturity Stages and Varieties Using Neural Networks Based on Volatile Organic Compounds
Source: Foods. 2025 Jan 8;14(2):169. doi: 10.3390/foods14020169 (PMC11765290; doi:10.3390/foods14020169)
Supplement: Supplementary file 1 [file foods-14-00169-s001.zip › foods-3392937-supplementary figures.pdf]

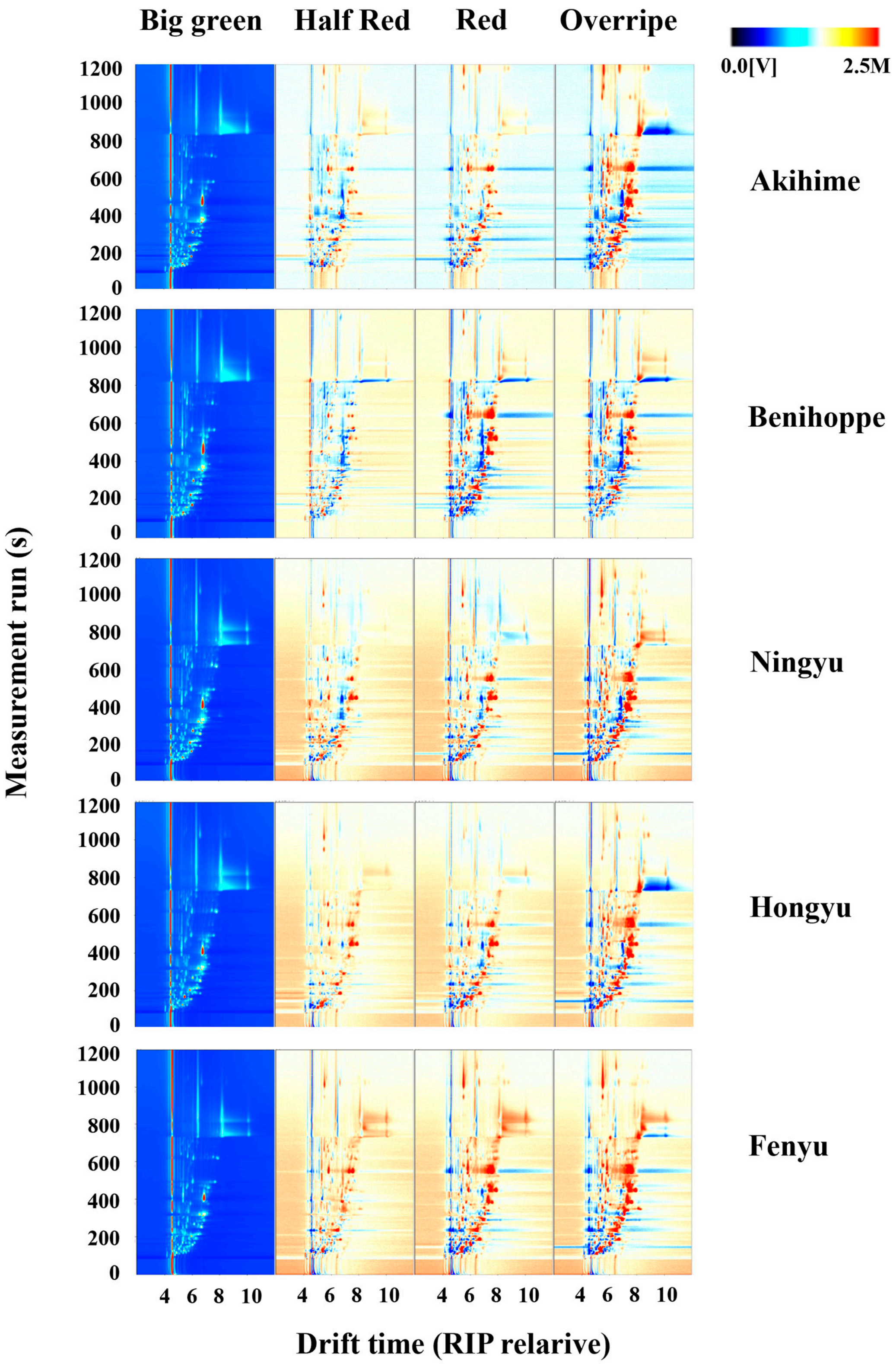

Figure S1: Spectrum comparison of the volatile compound profile of five strawberry varieties at four developmental stages. Big green stage was set as the reference plot. The color of the points represents the concentration of the substance (color bar), with white indicating identical concentration, blue indicating low concentration and red indicating high concentration.

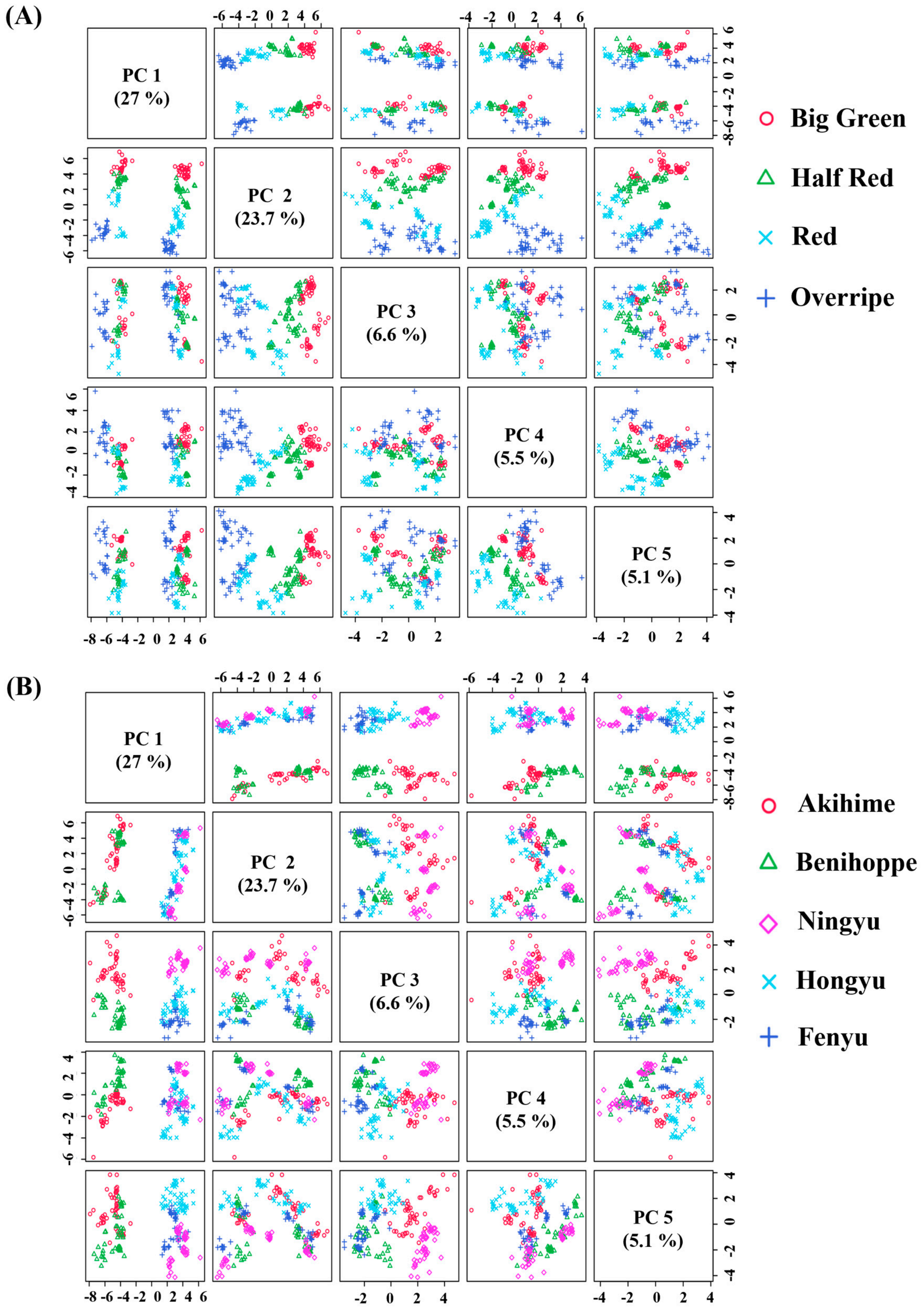

Figure S2: Principal component analysis (PCA) analysis from PC1 to PC5 of volatile components in four strawberry maturities (**A**) and five strawberry varieties (**B**)
